# Neurodivergence and mental health—Recognising what needs championing and challenging

**DOI:** 10.1371/journal.pmen.0000271

**Published:** 2025-03-17

**Authors:** Karli Montague-Cardoso, Luke Beardon, Amanda Kirby

**Affiliations:** 1 PLOS: Public Library of Science, Cambridge, United Kingdom; 2 Sheffield Hallam University, The Autism Centre, Sheffield, United Kingdom; 3 Do-IT Solutions Ltd, Cardiff, United Kingdom; PLOS: Public Library of Science, UNITED KINGDOM OF GREAT BRITAIN AND NORTHERN IRELAND

The beauty of humans is that every single one of us is unique. Neurodiversity embraces all our differences and refers to all of the different ways in which we move, communicate, act and process information [1]. Neurodivergence is a term used when that difference is considered to be beyond what has artificially been determined as ‘typical’. Neurodivergence and mental health are distinct but too often very much intertwined. As a journal, supporting the expansion of our understanding of the interactions between them, requires us to be inclusive of the plethora of perspectives and experiences of different neurodivergent communities and individuals. Whilst we can relate to others, we can never truly experience being someone else. We must listen to everyone and offer everyone a place at the table in whatever guise that may be. As a society, we need to strike a balance between not over-medicalising the experiences of neurodivergent communities, but at the same time, not trivializing them. Some individuals who identify as neurodivergent will embrace and celebrate their idiosyncrasies whilst others may feel that they endure struggles and obstacles in everyday life. Some individuals may indeed have both perspectives depending on their environment or circumstances and this may change over time as do our brains. Some individuals may be becoming more aware of the concept of neurodivergence and not yet know how they feel about this and are considering its meaning. They may not *want* to have to decide. And some may not be in a position in which an open exploration of what it means to be neurodivergent is even possible. But as a journal, if we strike the right balance and serve as a platform for all, we help to create a society in which all experiences are valued and all individuals are safe on their journey of self-discovery and what it means to be their authentic selves.

At *PLOS Mental Health*, our responsibility and ambition to represent the experiences of *all* neurodivergent communities in a balanced and sensitive way is largely dependent on the journal’s overall aim of taking every possible opportunity to accentuate and incorporate lived experience perspectives. This has been true since the conception of the journal, and will continue to receive our full attention - not just during this week, which is Neurodiversity Celebration Week, but on an interminable basis. Although the dialogue surrounding neurodivergence - and thus understanding - has come a long way over the last few years, we (academics, professionals, and society as a whole) still have a long way to go. Not only do we see vast regional and cultural differences in stigma, acceptance, understanding and practices, but we also see variations *between* different neurodivergent conditions and genders [2]. Every individual has their own unique characteristics and circumstances. Unless we can bring in the perspectives of as many individuals as possible, we will not be in a position to offer tailored information for those who may need or want it.

In order to aid the continuation of advances and acceptance, as well as help to steer reform where needed, not only do we need to be inclusive but we need to challenge existing practices and focuses - in terms of research, praxis and policies. We should not shy away from taking a critical stance and addressing the challenges that are really impacting day to day for many people...We must ask…What is working well? What is not working well? What has been overlooked? Where do existing biases and barriers come from? What remains inequitable and why?

To aid progress and thus enable us to challenge biases and barriers, we cannot overlook intersectionality. Intersectionality, which was proposed decades ago by Kimberle Crenshaw [3] refers to how our social and political identities, such as race and ‘class’, overlap, resulting in often compounding inequalities of various forms. Intersectionality recognises that, for instance, two women of different races in the same location, will experience sexism differently. In the context of neurodivergence, in 2019 the UK All-Party Parliamentary Group (APPG) released a report [4] that specifically acknowledged the intersections of gender and neurodiversity - stating that gender may affect individuals’ likelihood of receiving a diagnosis and/or support. On the subject of gender, it is also vitally important to point out that historically, most research on neurodivergence has been male-dominated [5]. But, it is increasingly (although slowly) being accepted that neurodivergent conditions such as Attention Deficit Hyperactivity Disorder (ADHD), Developmental Coordination Disorder (DCD, also known as Dyspraxia) and Autism Spectrum Conditions *can* be understood differently in males and females [6–8]. The prior overreliance on data from males has inevitably resulted in a bias lens when identifying and supporting whole communities. Likewise, there has been a significant imbalance in the focus of research, practice and policy, and therefore understanding of and support for, certain neurodivergent conditions. Though there are many reasons underlying this, the general focus on some traits and experiences as opposed to others is *not* based on prevalence in society [9]. For instance, Autism is much more commonly the subject of research questions and funding than dyslexia, despite the latter being reported more commonly, with an estimated 10% of the world’s population reported to be dyslexic relative to around 1.6% for Autism [10]. This is not to suggest of course that we should begin to research some characteristics more at the expense of others and we should certainly not approach conditions in isolation. We are simply calling for a more inclusive research landscape moving forward

In addition to the biases above, another factor that has been holding back our understanding of neurodivergence is a previously prevailing tendency for neurodivergent conditions to be considered in isolation - in other words - in the absence of co-existing traits or conditions. But in reality, we know it is very often not this simple and overlap or co-occurrence across conditions is common and therefore challenging the concepts of current diagnostic categorisations. By only considering ‘pure’ cases, we are missing so much of the person and increasing the likelihood of misdiagnosis and therefore sub-optimal support [11,12]. Failure to see a person as whole, who is influenced by their environment and its biases, will inevitably impact on wellbeing. However, whilst our appreciation for the interactions between neurodivergent traits and conditions and mental illness has increased, professional services continue to predominantly deliver mental health support separately. Ensuring that potential mental health needs are addressed simultaneously to the management of neurodivergent characteristics when needed requires a shift in research focus and practices, which first requires a shift in focus of research funding in the neurodiversity field towards wellbeing. Redirecting research funding efforts, along with publishers providing a platform for work that aims to eliminate biases and barriers, are both necessary for the field to progress effectively and meaningfully.

In keeping with the overarching mission of *PLOS Mental Health*, our ‘Neurodiversity and Mental Health Section’ aims to employ a combination of both championing and challenging approaches. During Neurodiversity Celebration Week and throughout 2025, the second year of *PLOS Mental Health*, we especially encourage submissions that support a more inclusive research landscape - considering experiences that have historically received less attention and exploring intersectionality in the context of neurodivergence, thus transforming research, practices and policies globally.
